# Supplementary material for: A Likelihood Approach for Real-Time Calibration of Stochastic Compartmental Epidemic Models
Source: PLoS Comput Biol. 2017 Jan 17;13(1):e1005257. doi: 10.1371/journal.pcbi.1005257 (PMC5240920; doi:10.1371/journal.pcbi.1005257)
Supplement: S1 File — (TAR.GZ) [file pcbi.1005257.s014.tar.gz › HSPH_Online-SI-Revision/Additions/IPoi10/n100-extreme-SEITR/n100-extreme-SEITR_BENCH_table0.pdf]

1	R0	{1.38654, 9.85565}	{2.97157, 19.3547}	{{2.97157, 19.3547}, {2.97157, 19.3547}}	{114.315, 96.3818}	inf	5261.	24 051.4	{17 263., 29 293.}	33.712	0.127476	newly	12 085.	8239.9	{5549., 10 586.}	31.8171	0.180785	Reff	0.312221	0.738924	{0.378637, 1.26018}	136.667	ar	92 379.	93 847.6	{93 756., 93 987.}	1.58979
2	R0	{2.36629, 12.2029}	{2.91946, 11.8546}	{{2.91946, 11.8546}, {2.91946, 11.8546}}	{23.3771, 2.85424}	inf	4672.	25 018.7	{16 232., 28 918.}	28.5344	0.158799	newly	17 453.	14 492.9	{11 365., 16 901.}	16.9603	0.0876495	Reff	0.202507	1.1043	{0.551749, 2.11775}	445.316	ar	99 022.	93 481.7	{93 344., 93 606.}	5.59502
3	R0	{2.32253, 12.836}	{2.98388, 9.60029}	{{2.98388, 9.60029}, {2.98388, 9.60029}}	{28.4754, 25.2081}	inf	6387.	19 550.8	{13 288., 26 823.}	46.138	0.282508	newly	18 266.	11 692.7	{7500., 16 554.}	37.5586	0.21953	Reff	0.29092	0.454914	{0.298686, 0.61265}	56.7801	ar	98 997.	93 931.6	{93 784., 94 072.}	5.11675
4	R0	{1.79075, 11.2058}	{2.82352, 10.8578}	{{2.80387, 10.6536}, {2.91946, 11.8546}}	{57.6725, 5.07435}	inf	6785.	19 837.6	{13 441., 27 032.}	29.2323	0.182414	newly	16 022.	10 979.1	{7140., 15 333.}	32.3386	0.198806	Reff	0.341174	0.584888	{0.358307, 0.874387}	72.0848	ar	96 920.	92 696.5	{92 371., 93 420.}	4.35776
5	R0	{2.26952, 9.62371}	{2.98388, 9.60029}	{{2.98388, 9.60029}, {2.98388, 9.60029}}	{31.4763, 0.243357}	inf	2810.	25 809.8	{20 853., 29 560.}	12.6624	0.0640602	newly	16 920.	17 032.8	{13 665., 21 073.}	14.6184	0.0645593	Reff	0.133902	0.836962	{0.493683, 1.50656}	525.057	ar	98 881.	93 948.3	{93 813., 94 100.}	4.98855
6	R0	{1.57191, 6.89004}	{2.9194, 10.1535}	{{2.9194, 10.1535}, {2.9194, 10.1535}}	{85.7231, 47.3649}	inf	4582.	23 409.7	{17 794., 28 732.}	21.3557	0.100028	newly	17 405.	14 826.4	{10 102., 18 987.}	19.1557	0.106091	Reff	0.240125	0.879511	{0.420043, 1.80264}	266.272	ar	95 046.	93 487.7	{93 340., 93 615.}	1.63955
7	R0	{1.99226, 17.2339}	{2.97157, 19.3547}	{{2.97157, 19.3547}, {2.97157, 19.3547}}	{49.1557, 12.306}	inf	5638.	23 868.	{17 558., 29 321.}	20.501	0.107747	newly	11 332.	8070.97	{5767., 10 442.}	28.7772	0.15702	Reff	0.270071	0.644758	{0.375072, 0.963829}	138.737	ar	97 992.	93 861.2	{93 705., 93 998.}	4.21549
8	R0	{2.63369, 15.1879}	{2.83858, 10.6973}	{{2.83858, 10.6973}, {2.83858, 10.6973}}	{7.77958, 29.567}	inf	3741.	23 135.7	{18 436., 27 457.}	37.138	0.207128	newly	14 766.	13 870.3	{10 136., 17 898.}	18.5947	0.0883472	Reff	0.159786	0.786778	{0.439611, 1.44492}	392.395	ar	99 451.	92 822.7	{92 640., 92 961.}	6.66493
9	R0	{2.53271, 9.90552}	{2.98388, 9.60029}	{{2.98388, 9.60029}, {2.98388, 9.60029}}	{17.8137, 3.08141}	inf	2374.	24 908.	{20 398., 29 247.}	20.5765	0.112246	newly	17 411.	17 074.7	{13 015., 21 344.}	16.904	0.0786222	Reff	0.11382	0.974556	{0.464978, 1.91434}	756.225	ar	99 355.	93 949.8	{93 852., 94 060.}	5.44027
10	R0	{2.48791, 18.9003}	{2.7943, 13.5962}	{{2.7943, 13.5962}, {2.7943, 13.5962}}	{12.3152, 28.0636}	inf	6612.	20 202.1	{13 447., 26 382.}	47.4874	0.293892	newly	13 614.	9533.59	{5986., 13 590.}	30.2042	0.1745	Reff	0.296509	0.669427	{0.354764, 1.37074}	125.77	ar	99 291.	92 436.9	{92 264., 92 585.}	6.90302
11	R0	{2.84414, 18.4765}	{2.99911, 15.6207}	{{2.99911, 15.6207}, {2.99911, 15.6207}}	{5.44875, 15.4564}	inf	5335.	24 268.6	{17 468., 29 976.}	42.1433	0.250284	newly	14 675.	10 243.5	{7392., 13 276.}	30.1977	0.168838	Reff	0.221814	0.800961	{0.397322, 1.36298}	261.095	ar	99 654.	94 042.7	{93 907., 94 218.}	5.63079
12	R0	{2.29369, 14.622}	{2.99939, 13.7076}	{{2.99939, 13.7076}, {2.99939, 13.7076}}	{30.767, 6.25359}	inf	4308.	24 925.4	{19 223., 29 728.}	25.0251	0.140851	newly	14 159.	11 740.	{8394., 14 529.}	18.5992	0.100965	Reff	0.188656	0.782209	{0.412446, 1.18761}	314.622	ar	98 982.	94 059.	{93 900., 94 192.}	4.97366
13	R0	{2.73413, 16.7529}	{2.9884, 12.8326}	{{2.9884, 12.8326}, {2.9884, 12.8326}}	{9.29985, 23.4007}	inf	4068.	22 498.8	{16 422., 28 686.}	42.3949	0.251963	newly	14 704.	11 025.9	{7548., 15 473.}	26.9826	0.148497	Reff	0.173699	0.636531	{0.354544, 1.08207}	266.456	ar	99 543.	93 941.4	{93 786., 94 086.}	5.62736
14	R0	{1.79554, 16.5943}	{2.86095, 19.9929}	{{2.86095, 19.9929}, {2.86095, 19.9929}}	{59.3365, 20.4805}	inf	7555.	20 821.8	{14 484., 27 803.}	26.2935	0.147848	newly	11 642.	6965.69	{4577., 9577.}	40.1676	0.240626	Reff	0.390135	0.749023	{0.358134, 1.56377}	95.7643	ar	96 821.	93 023.5	{92 875., 93 182.}	3.92222
15	R0	{1.91429, 13.7771}	{2.97157, 19.3547}	{{2.97157, 19.3547}, {2.97157, 19.3547}}	{55.2309, 40.4846}	inf	5519.	24 430.6	{17 743., 29 497.}	15.5837	0.0847395	newly	13 230.	8306.55	{5742., 10 427.}	37.2143	0.214217	Reff	0.264038	0.701428	{0.405055, 1.08878}	165.654	ar	97 541.	93 840.7	{93 653., 93 963.}	3.79357
16	R0	{1.42094, 10.5871}	{2.86118, 15.5722}	{{2.86118, 15.5722}, {2.86118, 15.5722}}	{101.358, 47.0865}	inf	7009.	19 537.8	{13 681., 26 424.}	20.339	0.0934909	newly	12 953.	8117.96	{5311., 11 997.}	37.3276	0.220815	Reff	0.417131	0.620262	{0.336618, 1.31752}	59.4106	ar	93 044.	93 009.4	{92 847., 93 178.}	0.111714
17	R0	{1.45515, 6.16466}	{2.98388, 9.60029}	{{2.98388, 9.60029}, {2.98388, 9.60029}}	{105.057, 55.7311}	inf	6217.	25 798.1	{21 612., 29 610.}	27.6021	0.105493	newly	20 574.	17 386.3	{13 495., 21 036.}	16.7434	0.0856406	Reff	0.356002	0.906831	{0.487238, 1.53804}	154.726	ar	93 254.	93 945.1	{93 780., 94 102.}	0.741075
18	R0	{2.85669, 13.2416}	{2.9194, 10.1535}	{{2.9194, 10.1535}, {2.9194, 10.1535}}	{2.1952, 23.3212}	inf	3137.	23 851.9	{18 284., 28 631.}	37.7869	0.215105	newly	17 189.	14 832.	{10 588., 19 365.}	18.9255	0.0954203	Reff	0.132179	0.785922	{0.412073, 1.46907}	494.589	ar	99 644.	93 484.8	{93 327., 93 645.}	6.18124
19	R0	{2.56417, 17.9558}	{2.86118, 15.5722}	{{2.86118, 15.5722}, {2.86118, 15.5722}}	{11.5831, 13.2748}	inf	6398.	24 824.5	{20 233., 28 275.}	37.2692	0.214132	newly	14 542.	10 671.1	{8187., 12 630.}	26.6186	0.144957	Reff	0.282084	0.881967	{0.486057, 1.30664}	212.661	ar	99 389.	93 028.6	{92 861., 93 199.}	6.39948
20	R0	{2.27517, 11.1864}	{2.9194, 10.1535}	{{2.9194, 10.1535}, {2.9194, 10.1535}}	{28.3157, 9.23353}	inf	3799.	23 415.2	{19 092., 28 324.}	26.7404	0.15826	newly	16 864.	14 350.6	{10 665., 18 921.}	19.6562	0.107168	Reff	0.172117	0.776829	{0.433735, 1.37361}	351.339	ar	98 894.	93 480.4	{93 311., 93 646.}	5.47412
21	R0	{2.35735, 10.4735}	{2.98388, 9.60029}	{{2.98388, 9.60029}, {2.98388, 9.60029}}	{26.5777, 8.33733}	inf	3960.	24 716.1	{18 822., 29 378.}	25.9576	0.136829	newly	18 543.	16 523.8	{11 897., 20 907.}	15.7914	0.0781323	Reff	0.172039	0.873244	{0.426247, 1.80844}	407.583	ar	99 036.	93 943.6	{93 776., 94 090.}	5.14193
22	R0	{1.79551, 11.9179}	{2.99939, 13.7076}	{{2.99939, 13.7076}, {2.99939, 13.7076}}	{67.0495, 15.0169}	inf	5574.	18 795.2	{10 195., 28 536.}	32.2166	0.191535	newly	14 003.	8486.16	{4314., 14 167.}	40.483	0.257685	Reff	0.270045	0.473031	{0.263946, 0.818534}	76.2115	ar	97 033.	94 037.4	{93 887., 94 165.}	3.08722
23	R0	{1.23545, 6.31687}	{2.9884, 12.8326}	{{2.9884, 12.8326}, {2.9884, 12.8326}}	{141.888, 103.148}	inf	4400.	24 971.4	{19 977., 29 701.}	81.7032	0.258939	newly	13 260.	12 877.	{9308., 15 995.}	17.3137	0.0823209	Reff	0.306775	0.897875	{0.422112, 1.63465}	192.682	ar	88 739.	93 982.1	{93 834., 94 129.}	5.90847
24	R0	{2.24089, 10.1789}	{2.83858, 10.6973}	{{2.83858, 10.6973}, {2.83858, 10.6973}}	{26.672, 5.09289}	inf	5491.	23 693.7	{17 591., 27 844.}	29.8629	0.180624	newly	20 012.	15 716.5	{11 015., 18 074.}	21.4646	0.119346	Reff	0.242442	1.27301	{0.656422, 1.97301}	425.076	ar	98 868.	92 843.9	{92 681., 93 025.}	6.09312
25	R0	{1.57298, 10.2607}	{2.99911, 15.6207}	{{2.99911, 15.6207}, {2.99911, 15.6207}}	{90.6642, 52.2382}	inf	7361.	26 180.	{19 359., 29 875.}	21.0805	0.103349	newly	15 457.	11 523.3	{9665., 13 295.}	25.4494	0.142118	Reff	0.403627	1.09687	{0.583567, 2.02026}	171.938	ar	94 957.	94 053.7	{93 888., 94 248.}	0.951277
26	R0	{1.2154, 8.2576}	{2.8939, 16.8013}	{{2.8939, 16.8013}, {2.8939, 16.8013}}	{138.103, 103.465}	inf	5404.	25 038.4	{17 391., 28 473.}	70.6154	0.239405	newly	11 730.	10 232.1	{8165., 11 782.}	13.1464	0.075193	Reff	0.35796	1.05468	{0.550564, 1.75243}	194.638	ar	88 421.	93 258.7	{93 112., 93 462.}	5.47121
27	R0	{2.22841, 18.7983}	{2.8939, 16.8013}	{{2.8939, 16.8013}, {2.8939, 16.8013}}	{29.8639, 10.6233}	inf	5503.	20 803.6	{14 079., 27 874.}	38.9152	0.226634	newly	11 908.	7929.71	{5219., 11 166.}	33.4086	0.192672	Reff	0.242317	0.57605	{0.341393, 0.943411}	137.725	ar	98 756.	93 285.5	{93 127., 93 442.}	5.53943
28	R0	{1.27778, 5.36767}	{2.98388, 9.60029}	{{2.98388, 9.60029}, {2.98388, 9.60029}}	{133.521, 78.854}	inf	5294.	19 318.3	{14 201., 28 685.}	26.2082	0.0987577	newly	18 063.	11 624.6	{8181., 19 671.}	38.4699	0.224647	Reff	0.340107	0.464511	{0.318499, 0.873591}	41.0477	ar	89 843.	93 930.9	{93 796., 94 101.}	4.55
29	R0	{2.38042, 15.8901}	{2.9884, 12.8326}	{{2.9884, 12.8326}, {2.9884, 12.8326}}	{25.5409, 19.2415}	inf	6676.	16 397.6	{10 258., 24 946.}	55.9335	0.389991	newly	15 388.	7652.58	{4517., 11 759.}	50.5586	0.340098	Reff	0.307407	0.426753	{0.268568, 0.623589}	45.7955	ar	99 112.	93 965.6	{93 807., 94 133.}	5.19247
30	R0	{2.53188, 12.7607}	{2.9194, 10.1535}	{{2.9194, 10.1535}, {2.9194, 10.1535}}	{15.3056, 20.4315}	inf	7079.	22 935.7	{6761., 28 915.}	41.5234	0.310234	newly	19 690.	15 913.1	{7923., 19 705.}	19.3328	0.138924	Reff	0.319143	1.3564	{0.669594, 2.61032}	325.012	ar	99 317.	93 466.2	{93 306., 93 616.}	5.89099
31	R0	{1.24482, 10.5471}	{2.93978, 19.9817}	{{2.93978, 19.9817}, {2.93978, 19.9817}}	{136.161, 89.4521}	inf	5495.	23 128.9	{18 036., 28 936.}	50.1979	0.176333	newly	9931.	7633.6	{5723., 10 023.}	23.7653	0.129451	Reff	0.364384	0.707936	{0.405807, 1.13675}	94.8673	ar	89 097.	93 618.	{93 472., 93 777.}	5.0743
32	R0	{2.30446, 17.9607}	{2.95209, 14.037}	{{2.95209, 14.037}, {2.95209, 14.037}}	{28.1033, 21.846}	inf	6256.	23 902.7	{17 931., 29 219.}	33.1505	0.186777	newly	13 001.	10 916.	{7555., 14 291.}	20.2883	0.107301	Reff	0.270705	0.694022	{0.380997, 1.08253}	156.376	ar	99 028.	93 705.4	{93 563., 93 860.}	5.37482
33	R0	{2.31579, 11.8236}	{2.9751, 8.95585}	{{2.9751, 8.95585}, {2.9751, 8.95585}}	{28.4702, 24.2545}	inf	3005.	9730.36	{4660., 15 636.}	67.9532	0.540803	newly	15 166.	5916.71	{2757., 9517.}	63.3083	0.472632	Reff	0.14124	0.285965	{0.22102, 0.338299}	102.467	ar	99 012.	93 869.5	{93 720., 94 016.}	5.19386
34	R0	{1.6678, 10.6744}	{2.99939, 13.7076}	{{2.99939, 13.7076}, {2.99939, 13.7076}}	{79.8411, 28.4156}	inf	4965.	19 924.1	{13 815., 26 801.}	20.7138	0.104508	newly	13 645.	9049.54	{5998., 12 809.}	34.3209	0.196803	Reff	0.259643	0.533535	{0.307947, 0.742499}	105.488	ar	95 809.	94 045.8	{93 895., 94 168.}	1.84031
35	R0	{1.65476, 7.07559}	{2.98388, 9.60029}	{{2.98388, 9.60029}, {2.98388, 9.60029}}	{80.321, 35.6818}	inf	5229.	25 611.	{20 318., 29 796.}	17.5084	0.0758074	newly	19 217.	16 932.5	{11 899., 21 277.}	17.7771	0.0897494	Reff	0.270404	0.841734	{0.432126, 1.37366}	211.287	ar	95 746.	93 946.7	{93 826., 94 088.}	1.87928
36	R0	{2.71807, 18.5959}	{2.7943, 13.5962}	{{2.7943, 13.5962}, {2.7943, 13.5962}}	{2.80456, 26.886}	inf	7257.	23 366.8	{17 247., 27 524.}	44.1839	0.276684	newly	15 024.	12 056.1	{9509., 13 864.}	19.7543	0.110239	Reff	0.322961	1.18671	{0.617121, 1.9362}	267.447	ar	99 540.	92 472.2	{92 310., 92 609.}	7.10045
37	R0	{2.6202, 12.7552}	{2.99939, 13.7076}	{{2.99939, 13.7076}, {2.99939, 13.7076}}	{14.4718, 7.46676}	inf	5145.	26 288.	{20 728., 30 075.}	32.8206	0.192173	newly	18 951.	13 082.9	{10 883., 15 102.}	30.9648	0.173107	Reff	0.212734	1.07514	{0.565715, 1.94267}	405.394	ar	99 426.	94 044.	{93 902., 94 157.}	5.41303
38	R0	{1.47672, 9.81651}	{2.86118, 15.5722}	{{2.86118, 15.5722}, {2.86118, 15.5722}}	{93.7524, 58.6328}	inf	5442.	22 742.	{16 427., 28 126.}	21.743	0.0914216	newly	13 535.	9767.69	{6738., 12 460.}	27.8338	0.15238	Reff	0.299449	0.864392	{0.402482, 1.84131}	188.661	ar	93 815.	92 987.8	{92 836., 93 146.}	0.881785
39	R0	{1.90004, 12.3313}	{2.96307, 14.3219}	{{2.96307, 14.3219}, {2.96307, 14.3219}}	{55.9478, 16.1427}	inf	6237.	24 011.8	{19 663., 28 707.}	16.9721	0.0930207	newly	15 042.	11 049.8	{8310., 14 139.}	26.5404	0.147282	Reff	0.303056	0.821141	{0.425378, 1.64886}	171.04	ar	97 413.	93 776.4	{93 646., 93 924.}	3.73315
40	R0	{2.64218, 18.5237}	{2.9884, 12.8326}	{{2.9884, 12.8326}, {2.9884, 12.8326}}	{13.1036, 30.7233}	inf	4328.	17 618.6	{10 597., 25 464.}	53.47	0.356243	newly	13 194.	8236.14	{4855., 12 060.}	39.1801	0.239654	Reff	0.17626	0.4078	{0.271018, 0.57192}	131.363	ar	99 474.	93 962.4	{93 824., 94 091.}	5.5407
41	R0	{1.6945, 7.17996}	{2.9194, 10.1535}	{{2.9194, 10.1535}, {2.9194, 10.1535}}	{72.2868, 41.4144}	inf	7462.	22 972.5	{8067., 29 201.}	19.8853	0.141925	newly	23 349.	16 077.9	{9189., 19 632.}	31.1411	0.188244	Reff	0.379415	1.36568	{0.605921, 2.55144}	259.944	ar	96 332.	93 488.8	{93 329., 93 654.}	2.95143
42	R0	{1.84559, 18.0074}	{2.97157, 19.3547}	{{2.97157, 19.3547}, {2.97157, 19.3547}}	{61.0092, 7.48192}	inf	6919.	23 930.3	{17 954., 29 325.}	17.3474	0.0893055	newly	10 541.	8179.51	{5834., 10 567.}	22.6246	0.121541	Reff	0.338389	0.694556	{0.376825, 1.23906}	105.308	ar	97 358.	93 826.2	{93 690., 93 964.}	3.62765
43	R0	{2.24965, 17.0361}	{2.7943, 13.5962}	{{2.7943, 13.5962}, {2.7943, 13.5962}}	{24.2104, 20.1918}	inf	7490.	20 274.9	{13 300., 26 912.}	42.5836	0.253855	newly	14 237.	9350.43	{5807., 13 242.}	34.3231	0.199922	Reff	0.339337	0.574915	{0.362393, 0.904235}	70.0302	ar	98 770.	92 440.8	{92 309., 92 583.}	6.40807
44	R0	{2.54324, 15.7967}	{2.95209, 14.037}	{{2.95209, 14.037}, {2.95209, 14.037}}	{16.076, 11.1397}	inf	3895.	25 553.1	{19 857., 29 481.}	29.5067	0.162088	newly	14 170.	12 270.4	{9191., 14 484.}	14.3371	0.0735813	Reff	0.167193	0.977829	{0.483405, 1.89264}	484.852	ar	99 354.	93 719.7	{93 567., 93 865.}	5.67093
45	R0	{2.54943, 14.8032}	{2.83858, 10.6973}	{{2.83858, 10.6973}, {2.83858, 10.6973}}	{11.3418, 27.7366}	inf	4134.	22 937.1	{17 320., 27 513.}	36.7741	0.218075	newly	14 996.	13 930.6	{9876., 17 594.}	17.1585	0.0890449	Reff	0.179352	0.881543	{0.427547, 1.83696}	391.514	ar	99 380.	92 821.8	{92 653., 92 994.}	6.59911
46	R0	{2.37755, 12.6461}	{2.98388, 9.60029}	{{2.98388, 9.60029}, {2.98388, 9.60029}}	{25.5023, 24.085}	inf	5896.	13 702.9	{7983., 22 964.}	62.2062	0.458362	newly	17 991.	8035.48	{4383., 13 604.}	56.245	0.398477	Reff	0.261673	0.348912	{0.240322, 0.476824}	36.7473	ar	99 096.	93 941.	{93 772., 94 076.}	5.20198
47	R0	{1.69279, 11.6636}	{2.95376, 15.278}	{{2.95376, 15.278}, {2.95376, 15.278}}	{74.4906, 30.9887}	inf	6849.	24 466.7	{18 213., 29 260.}	14.2867	0.0778822	newly	14 547.	10 509.2	{7205., 13 270.}	27.7567	0.158522	Reff	0.3499	0.786249	{0.407087, 1.23157}	124.707	ar	96 214.	93 733.2	{93 599., 93 882.}	2.57846
48	R0	{1.82402, 10.0038}	{2.9884, 12.8326}	{{2.9884, 12.8326}, {2.9884, 12.8326}}	{63.8359, 28.2773}	inf	6918.	25 841.1	{20 772., 29 696.}	11.775	0.0700462	newly	17 877.	13 125.4	{9683., 15 802.}	26.5796	0.14799	Reff	0.343281	0.86013	{0.449963, 1.26385}	150.562	ar	97 076.	93 961.5	{93 810., 94 117.}	3.20833
49	R0	{1.6424, 11.1667}	{2.99939, 13.7076}	{{2.99939, 13.7076}, {2.99939, 13.7076}}	{82.6224, 22.7543}	inf	4677.	17 185.3	{11 079., 24 668.}	29.0332	0.161745	newly	12 499.	7642.34	{4763., 11 279.}	40.359	0.24743	Reff	0.249793	0.418096	{0.273484, 0.544869}	67.5722	ar	95 546.	94 022.	{93 865., 94 141.}	1.59509
50	R0	{2.05638, 14.3048}	{2.9884, 12.8326}	{{2.9884, 12.8326}, {2.9884, 12.8326}}	{45.3233, 10.2917}	inf	6276.	22 743.	{15 452., 29 612.}	28.0104	0.161927	newly	14 245.	11 148.5	{7121., 15 624.}	24.4294	0.134866	Reff	0.290299	0.67755	{0.343786, 1.03494}	133.397	ar	98 194.	93 982.5	{93 835., 94 136.}	4.289
